# Teleneuropsychology: Reliability and Acceptance in Memory Assessment

**DOI:** 10.1089/tmr.2024.0066

**Published:** 2024-12-24

**Authors:** Mariana Varandas, Filipa Ribeiro

**Affiliations:** ^1^ Universidade Católica Portuguesa, Institute of Health Sciences, Lisboa, Portugal.; ^2^ Universidade Católica Portuguesa, Institute of Health Sciences, Center for Interdisciplinary Research in Health, Portugal.

**Keywords:** episodic memory, neuropsychological tests, telehealth, teleneuropsychology

## Abstract

**Introduction:**

The use of telehealth in psychological interventions has experienced a significant increase in recent years. This form of patient interaction has important implications, especially in neuropsychological assessment. Given the limited research on this subject in Portugal, the reliability of neuropsychological tests and acceptance of this format by the Portuguese elderly must be studied.

**Methods:**

The sample consisted of 43 healthy Portuguese adults. They were assessed by cognitive measures and questionnaires regarding sociodemographic aspects, memory complaints, computer literacy, functionality, depressive symptoms, and satisfaction with the teleconsultation via videoconference and in-person approaches. The order of the two conditions was randomized.

**Results:**

There were no significant differences between scores on the Logical Memory Test. However, higher scores were found in the online condition for the Paired Associate Learning Test. The level of satisfaction with teleconsultation was high, but it did not translate into a greater preference for this format.

**Discussion:**

The neuropsychological assessment of auditory-verbal episodic memory through teleconsultation is promising and well accepted by the older Portuguese population. The data indicate that teleconsultation provides reliable results for episodic memory assessment.

## Introduction

The COVID-19 emergency and the need to minimize the risk of contamination led to an exceptional spread of telehealth in Portugal in 2020. Telehealth prevented the health system from collapsing and enabled the monitoring of chronic patients.^1^ Beyond pandemics, telehealth brings advantages in other public health emergency scenarios, like annual flu peaks. Telehealth also has the potential to reduce travel costs, time for outpatient appointments, and waiting lists. Therefore, even after the COVID-19 pandemic, it “seems to have the potential to become a common practice in the future of healthcare in Portugal.”^2^

This trend is also seen in psychology, with telepsychology offering psychological services such as counseling, intervention programs, and assessments through telecommunication technologies.^3^ Specifically, teleneuropsychology could include neuropsychological assessment or intervention delivered via telehealth. Regarding client acceptance of teleneuropsychology, a study by Appleman et al. with American veterans suggested a 90% satisfaction rate, as reported in a face-to-face condition,^4^ rising to 98% in a study by Parikh et al.^5^ They found that around two-thirds of the participants had no preference or would prefer a teleassessment.^5^ A Portuguese study monitored a telehealth project that included more than 100,000 teleconsultations of several specialties, including neurology but not psychology, between 1998 and 2011. The project obtained a 96% satisfaction rate from both patients and health care professionals and was based in Alentejo, a Portuguese region characterized by an isolated and aging population with low levels of formal education.^6^

When COVID-19 began, the American Psychological Association (APA) released guidelines for teleassessment. These guidelines helped monitor the quality of the collected data and ensured ethical and deontological standards.^7^ Likewise, the Inter Organizational Practice Committee (IOPC) also made recommendations. Before the session, the neuropsychologist must identify eligible participants for this format, negotiate where teleconsultation will take place, and, if necessary, identify a person to assist the patient with device interaction. During teleassessments, the professional must pay attention to the patient’s nonverbal behavior, provide alternatives in case of technical difficulties, and share the screen to ensure images of the highest quality.^8^

Teleconsultation brings new challenges to psychologist–patient interactions, either technological ones like poor audio/video quality, difficulties maintaining connection, and discomfort handling equipment, or relational ones such as greater psychological distance and distortion of eye contact.^4,9^ For the neuropsychologist, important details such as tremor, fatigue, appearance, or hygiene are harder to identify through a screen. Also, the self-efficacy beliefs regarding technology may lead to low motivation or anxiety.^10^ Such contextual differences can influence the psychologist and patient interactions, impacting the patient’s performance and subsequent results.

Studies of equivalence between in-person assessment and teleassessment have reached different conclusions, depending on the protocols and the samples used. Several studies showed comparable results in both conditions in the subtests of the Repeatable Battery for the Assessment of Neuropsychological Status (RBANS),^11^ the Mini-Mental State Examination (MMSE),^12^ and in verbal fluency tests and only minor differences in the Digit Span.^13^ In other studies, the MMSE screening test and the Clock Drawing Test proved significantly lower in teleconsultation,^9^ but the Rey Auditory Verbal Learning Test showed higher in the online condition.^14^

Since episodic memory is crucial in neuropsychological assessments in the elderly,^15^ it is essential to evaluate the reliability of teleassessment for commonly used memory tests such as the Word Pairs Associates and the Logical Memory Tests.^16^ Rizzi et al.^17^ sought to create Italian normative data for episodic memory tests through teleassessment. Their results indicated reliable scores in the two conditions for the Word Pairs Associates Test with high agreement coefficients.^17^ Regarding the Logical Memory Test, Mitsis et al. also reported statistically equivalent scores when applying this test via telephone or in-person to a sample of females aged over 65.^18^ In contrast, Jacobsen et al. and Krynicki et al. found higher mean scores in the teleassessment than in the in-person assessment for the Logical Memory Test.^19,20^

Although most studies opt for an intrasubject design, comparing the results obtained in both forms of assessment, this design raises questions related to the practice effect, so an intergroup comparison design should also be considered. Other important aspects tend to vary in the research, such as the sample size and characteristics, days between sessions, equipment used, the presence of a second professional during the teleconsultation, and the type of stimuli and output required. A meta-analysis of 12 studies utilizing a counterbalanced crossover design demonstrated similar performance in face-to-face and online conditions in studies with participants aged 65 and 75 years old, using verbal tests and high-speed network connections. However, conclusions are less consistent when participants are older, internet connections are slower, and when the tests have visual or motor components.^21^ Other systematic reviews^10,22^ also supported the use of screening tests, such as MMSE, both for healthy individuals and those with dementia. However, they recognized the lack of support for equivalence between the two conditions for the Clock Drawing Test and measures of executive functions.

Teleassessment for neuropsychological evaluations is generally reliable for various instruments and patients. However, it is important to investigate its effectiveness in Portugal, where an aging population with low computer literacy could raise concerns and lead to different results than those observed in other countries.^23^ In the only Portuguese study, a screening test was administered in-person, through telephone, and by videoconference to elderly people. There was a strong association between the results in the three conditions.^24^ No further studies are known, so it could prove interesting to study the reliability of neuropsychological teleassessment in Portugal, with older participants, using other neuropsychological tests. The present study aims to address this need, assessing both the reliability of verbal episodic memory tests by teleassessment and the acceptance of this format for the Portuguese elderly.

## Methods

### Participants

The participants were volunteers from Mafra, Lisbon, who were either attending a senior university or participating in an active aging program. The inclusion criteria were being over 55 years old and having a Montreal Cognitive Assessment (MoCA)^25^ score above the cutoff point for age and education^26^ (suggesting no evidence of cognitive deterioration). This instrument is a widely used cognitive screening tool designed to detect mild cognitive impairment by evaluating memory, attention, language, and executive functions. The following were the exclusion criteria: presence of diagnosed neurological diseases, inability to speak/understand Portuguese fluently, presence of uncorrected visual or hearing difficulties, and Instrumental Activities of Daily Living Questionnaire (IADL) score above the cutoff point (indicating reduced ability to perform everyday activities).^27^

### Procedures

This study was submitted for approval to the Scientific Council and the Ethics Committee of the Institute of Health Sciences at the Universidade Católica Portuguesa.

All participants provided informed consent before any procedures were carried out. A semi-structured interview recorded the demographic status, clinical information, neurological and psychiatric history, and computer literacy level.

The following instruments were administered to participants who met the inclusion and exclusion criteria: the Subjective Memory Complaints Questionnaire (SMCQ),^28^ the Word Pairs Associates Test and the Logical Memory Test from the Bateria de Lisboa de Avaliação de Demências (BLAD),^29^ the Geriatric Depression Scale (GDS-15),^30^ and a Satisfaction with Videoconference Administration Questionnaire.

The SMCQ^28^ is a tool designed to evaluate memory complaints and their severity. It comprises ten multiple-choice questions that are based on the Likert scale. These questions are related to concerns such as memory, language, orientation, slowness, and the ability to concentrate. Scores above 4 indicate the presence of subjective memory complaints that hold some importance. This result may suggest that there is a real deficit.

The Word Pairs Associates Test^16,29^ is a widely used test in neuropsychological examinations of episodic memory and associative learning. The test consists of 10 pairs of words, which are orally presented by the examiner. After reading out the list, the examiner presents the first word of each pair again, and the examinee needs to complete the pair. This task is repeated three times. After 30 min, there is a delayed recall task, in which the examiner presents the first word of each pair, and the examinee must associate the correct word with it. One point is given for each correctly associated word.

The Logical Memory Test^16,29^ includes two short stories (A and B) that are presented orally. After the examiner finishes reading each story, the examinee is asked to recall everything they remember from the story. After a break of ∼30 min, the participant is asked to remember each of the stories once again. One point is given for each answer corresponding to each of the story units, and no points are awarded if it does not meet the quotation criteria. The result corresponds to the average of the ideas evoked in the two stories. The quotation for the first part (immediate recall) is made separately from the quotation for the second part (delayed recall). It is also possible to calculate the rate of information lost between these two moments, characterized by the difference between delayed recall and immediate recall (forgetting measure).

The GDS is a self-report tool used to assess depressive symptoms in elderly individuals. For this study, the short form of 15 items was used.^30^

To determine the level of computer literacy, a Likert-type question, “How comfortable do you feel using new technologies?” was asked. Participants rated their comfort level on a scale of 1 (not comfortable at all) to 5 (very comfortable). This question was presented at the end of the online session, regardless of whether it was the first evaluation or not.

Satisfaction with Videoconference Questionnaire aims to assess participants’ satisfaction and likelihood of undergoing another neuropsychological assessment through multiple-choice and 5-point Likert scale questions. They were considered answers to the following questions: “Did you experience any issues regarding the quality of the video or audio?” “What device did you use during the teleconsultation?” “What is your overall satisfaction of this teleassessment?” and “Which of the two assessment conditions do you prefer?”

Every participant completed two sessions, one in-person and one online, with the order randomized. The face-to-face session lasted one hour in a quiet room in the institution where participants were recruited. The online session, lasting 40 min, was conducted via Zoom or Google Meet. At the start of the face-to-face session, participants completed the MoCA test and a question regarding computer literacy level. The SMCQ was completed in the first session regardless of the consultation format. Subsequently, the Word Pairs Associates Test and Logical Memory Test were presented. During the delay between immediate and delayed recall, either the GDS-15 or the IADL was also administered. At the end of the online session, participants completed the Satisfaction Questionnaire.

In total, participants completed the Word Pairs Associates Test twice (one per session) and the Logical Memory Test once (either in-person or online) to prevent the practice effect. Practice effects occur when repeated testing artificially improves performance due to familiarity with the material, rather than genuine cognitive improvement,^31^ and according to literature, Logical Memory tests are particularly prone to this effect.^32^

The teleconsultation was conducted using either the participants’ own devices, at home, or, if there were none available, on devices provided by the senior university. Assistance was provided to participants who struggled with device setup. Therefore, the devices used were those accessible for common use and were not selected by screen size, resolution, or sound system. Participants were sent an email before the online session containing a link to access the meeting and a recommendation to select a quiet location with a stable internet connection. All self-report questionnaires and visual aids were presented to the participants via screen sharing during the online session.

## Results

Forty-three subjects (79.10% female) with no cognitive deterioration were included in this study. Demographic data are shown in Table 1. The computer literacy perception mean was 3.67 (SD = 1.16), mode 4. The number of days between the two sessions was on average 6.42 days (SD = 3.97). Most participants (88.4%) used a computer, and the rest used a tablet device. 25.58% of sessions presented image interruptions (<2 sec) due to the quality of the connection. There was no evidence of a significant association between the device and the technical difficulties reported (Φ = 0.120, *p* = 0.432).

**Table 1.** Sample Characterization

	Mean	SD	Min–Max
Age	69.72	7.14	57–87
Years of formal education	12.16	4.80	1–23
MoCA	25.93	2.13	20–29
GDS-15	3.35	3.12	0–9
SMCQ	5.56	3.08	0–11
IADL	8	0	8
Computer literacy^a^	3.67	1.17	1–5
Teleconsultation satisfaction^b^	4.56	0.63	2–5

^a^ Reported based on responses to the question, “In general, how comfortable you feel using technology?”

^b^ Reported based on responses to the question, “What is your overall satisfaction of this teleassessment?”

GDS-15, Geriatric Depression Scale (short form); IADL, Instrumental Activities of Daily Living; MoCA, Montreal Cognitive Assessment; SMCQ, Subjective Memory Complaints Questionnaire; SD, standard deviation.

For an intersubject comparison, the sample was divided into two groups based on the format of the first session. Twenty-two participants completed their first session in-person and 21 via videoconference. No significant differences were detected, according to age, education, subjective memory complaints, computer literacy, depressive symptoms, screening test, or sex (χ^2^[1, *N* = 43] = 0.206, *p* = 0.650; Table 2).

**Table 2.** Descriptive Statistics and *t*-Test of the Groups Characteristics and Performance According to the Administration Format of Logical Memory Test

	In-person *N* = 21		Online *N* = 22			
	M	SD	M	SD	*t*	*p*
Age	68.809	7.850	70.591	6.463	−0.814	0.420
Education	11.714	5.139	12.590	4.532	−0.594	0.556
SMCQ	5.476	3.516	5.636	2.682	−0.168	0.867
Computer literacy^a^	3.619	1.161	3.727	1.202	−0.300	0.766
GDS	3.619	3.138	3.091	3.161	0.550	0.586
MoCA	25.381	2.085	26.455	2.087	−1.687	0.099
BLAD A Story						
Immediate recall	14.095	3.740	14.409	3.621	−0.280	0.781
Delayed recall	13.00	4.289	13.182	4.584	−0.134	0.894
BLAD B Story						
Immediate recall	9.191	3.040	9.909	2.505	−0.847	0.402
Delayed recall	8.143	2.071	9.318	3.483	−1.188	0.242
Composite BLAD^b^						
Immediate recall	11.643	2.937	12.159	2.607	−0.610	0.545
Delayed recall	10.571	3.195	11.250	3.572	−0.656	0.516
Forgetting measures^c^						
BLAD A	−1.095	1.895	−1.227	2.409	0.199	0.422
BLAD B	−1.048	2.224	−0.591	1.943	−0.718	0.238

^a^ Reported based on responses to the question: “In general, how comfortable you feel using technology?”

^b^ Arithmetic mean of the scores for A and B.

^c^ Difference between the delayed recall score and the immediate recall score.

BLAD, Bateria de Lisboa de Avaliação de Demências.

Performances on the Logical Memory Test were compared between in-person and online conditions using a *t*-test for independent samples. There were no significant differences in the immediate, delayed recall, and the forgetting measures in the two assessment conditions (Table 2).

Performances of the Word Pairs Associates Test were compared using a mixed repeated measures analysis of variance, considering the within-subject factor as the administration number (first or second) and between-subject factor as the administration format (in-person and online). There was a significant effect of administration number (*F*[1, 41] = 34.154, *p* < 0.001, η^2^p = 0.419), thereby participants recalled more information in the second administration (M = 18.69; SD = 0.39; mean difference = 2.135, *p* < 0.001). A significant effect of the first administration format (*F*[1, 41] = 4.590, *p* = 0.038, η^2^p = 0.097) was also found with the participants recalling more information in the online condition (M = 18.39; SD = 0.51; mean difference = 1.529, *p* = 0.04). Although there was not an interaction effect between first administration format and administration number (*F*[1, 41] = 0.753, *p* = 0.391).

Overall teleconsultation satisfaction averaged 4.56 (SD = 0.63) with a mode of 5, referred to the 5-point Likert scale question, “What is your overall satisfaction of this teleassessment?” There was also no evidence of a significant association between satisfaction and technical difficulties (χ^2^[2, *N* = 43] = 0.371, *p* = 0.831, Cramer’s *V* = 0.093) nor perception of computer literacy (*ρ*[41] = 0.102; *p* = 0.516). Whether this format was the first evaluation or not, satisfaction was not affected (χ^2^[2, *N* = 43] = 1.381, *p* = 0.501, Cramer’s *V* = 0.179). Despite the high level of satisfaction, no significant association between satisfaction and preference for neuropsychological assessment format was found (χ^2^[4, *N* = 43] = 3.891, *p* = 0.421, Cramer’s *V* = 0.213). More than half of the participants (58.10%) expressed a preference for undergoing new neuropsychological assessments in-person and 32.60% had no preference. Only 9.30% of the participants preferred undergoing new neuropsychological assessments online. Despite this, a significant association was found between the preference for administration mode and the perception of computer literacy (χ^2^[8, *N* = 43] = 17.401, *p* = 0.026, Cramer’s *V* = 0.450). Henceforth, participants with greater computer literacy were more likely to prefer taking an assessment in-person or had no preference.

## Discussion

The purpose of this study was to analyze the reliability of memory tests via teleconsultation among older adults in Portugal. With the increased use of teleconsultation in neuropsychological assessments, it is crucial to examine its implications and overall acceptance among the Portuguese elderly population, considering the limited research available on this topic.

The participants undertook two neuropsychological memory tests in both face-to-face and online formats. Results from two well-known neuropsychological tests were investigated, one employing intersubject comparisons and the other using an intrasubject comparison, which can confirm a practice effect. For the Logical Memory Test, we compared the results from the group administered the test online with those administered in-person. Since the administration of the Word Pairs Associates Test occurred in both sessions, these results were compared by a repeated measures analysis.

Our results suggest that there are no significant differences in the raw results of the Logical Memory stories at both recalls between online and in-person conditions, in line with findings reported in the literature, particularly by Mitsis et al.^18^ Likewise, the amount of information lost during latency was not influenced by the assessment format. However, these conclusions contradict findings from other studies,^19–20^ as they reported better scores on the Logical Memory Test during online sessions. Notably, the participants in those studies were younger than those in the present study, and as this paper suggests, age may influence computer literacy and comfort with teleconsultation technology. This age-related factor could impact assessment anxiety, making younger participants feel more comfortable and focused on tasks when using a mobile device.^18^ Even so, the current results suggest that the means of test administration do not significantly influence the Portuguese elderly’s ability to retrieve stories presented auditorily after their presentation or following a break.

Scores in the Word Pairs Associates Test were higher in the online format. This finding was unexpected and inconsistent with a recent study by Rizzi et al.^17^ which, although conducted with a younger sample, revealed high agreement between conditions. However, our differences were minor, and although they may have statistical significance, they may not be clinically relevant in interpreting the diagnosis. Some studies that analyzed scores from other auditory-verbal memory tests have found similar results advocated low distraction perception during testing via telecommunication because of the absence of an examiner at the same location.^18^ As expected, a practice effect was also found.

The teleconsultation satisfaction of participants was high and consistent with previous studies, such as the Portuguese one.^18^ However, this high level of satisfaction did not translate into a strong preference for teleconsultation over in-person assessments. Our sample preferred conventional in-person assessment. It is possible that people’s conservationism and the fact that the elderly Portuguese population has only recently begun having greater contact with technologies—due to COVID-19—may be contributing to this preference. While participants expressed satisfaction with the teleconsultation experience, many were not eager to repeat it. It is possible that the responses provided by some participants may have been influenced by social desirability bias. This means that they may have answered in a way that they thought would please the researcher, for example, by selecting the highest score in “What is your overall satisfaction of this teleassessment?” or by choosing the conventional mode (in-person) or not expressing a stance (no preference) in “Which of the two assessment conditions do you prefer?”^33^

It was surprising that even participants with lower levels of computer literacy expressed satisfaction with teleassessment. Support was offered to those who admitted difficulties with computers, hence enabling them to participate in the online task with the help of a senior university secretary, following IOPC recommendations. On the other hand, participants with greater computer literacy accessed the session independently, potentially managing unexpected issues. As a result, those with lower computer literacy were shielded from the challenges of technology, leading to a positive experience, while those with higher literacy were more aware of potential technical limitations.

Overall, the findings indicate that teleservice-based neuropsychological assessment of auditory-verbal episodic memory is promising and should be well received by older Portuguese adults.

Thus, Portugal should continue to invest in the digital transformation of its health care institutions, training health professionals, and improving computer literacy for citizens. It is important to analyze who this format of assessment would be beneficial for, which cognitive functions can be tested, and with which tests. Additionally, recommendations from recognized institutions such as the APA and IOPC should be considered in terms of providing informed consent and ensuring the privacy and security of assessment instruments, as well as the quality of interaction with the patient and data collection.

Despite the positive findings, certain limitations make generalization difficult. Although we opted for an intrasubject analysis of the Logical Memory Test, a within-subject analysis (comparing the results of both in-person and online administrations) would have been preferable to minimize error. Even though the Logical Memory Test is particularly vulnerable to the practice effect,^32^ it is important to conduct further intrasubject analyses on the Logical Memory Test within larger samples and with a longer time gap between sessions to minimize the learning effect. Furthermore, the sample consisted mainly of highly educated elderly individuals, meaning that the results may not be representative of the broader Portuguese elderly population, especially those with lower educational levels.

Future studies should continue to accumulate evidence on teleassessment. Extending the scope of this study to include elderly individuals of varying ages, education, and computer literacy is crucial. It would also be beneficial to include individuals with neuropsychiatric diagnoses, as previous research has shown inconsistent equivalence between in-person and online assessments in older or hospitalized populations.^9,21^ It would be possible to assess behavior, satisfaction, and acceptance of the clinical population during the session and study diagnostic agreement. It would also be beneficial to incorporate a wider range of neuropsychological tests, including those that require nonverbal responses, as these tend to be more affected by online testing conditions. Finally, in the future, a more extensive questionnaire regarding environmental factors of teleconsultation and intrinsic factors of the patient should be reported. The reports of levels of state anxiety and distraction perception in both conditions could corroborate the assumption about the low distraction perception during online testing.

## Conclusion

This study demonstrates that a sample of Portuguese elderly individuals exhibited a similar performance pattern of results or minor differences with low clinical significance in two widely used verbal episodic memory tests administered either face-to-face or via videoconference. It is a relevant contribution with results that support the hypothesis of the reliability and good acceptance of neuropsychological assessment by videoconference in a sample of Portuguese elderly people.

## Abbreviations Used

APA: American Psychological Association
BLAD: Bateria de Lisboa de Avaliação de Demências
GDS-15: Geriatric Depression Scale
IADL: Instrumental Activities of Daily Living Questionnaire
IOPC: Inter Organizational Practice Committee
MMSE: Mini-Mental State Examination
MoCA: Montreal Cognitive Assessment
RAVL: Rey Auditory Verbal Learning Test
RBANS: Repeatable Battery for the Assessment of Neuropsychological Status
SD: Standard Deviation
SMCQ: Subjective Memory Complaints Questionnaire
